# Association of polymorphisms of renin angiotensin system and endothelial nitric oxide synthase genes with premature cardiovascular disease in an Iranian population

**DOI:** 10.1186/s12872-023-03276-x

**Published:** 2023-05-16

**Authors:** Hoorak Poorzand, Bahareh Fazeli, Omid Khajavi, Arash Gholoobi, Faeze Keihanian, Negar Morovatdar

**Affiliations:** 1 Vascular and Endovascular surgery research center, Mashhad University of Medical Sciences, Mashhad, Mashhad, Iran; 2 Inflammation and Inflammatory Diseases Research Center, Mashhad University of Medical Sciences, Mashhad, Iran; 3 Cardiovascular Department, Faculty of Medicine, Mashhad University of Medical Sciences, Mashhad, Iran; 4 Clinical Research Unit, Faculty of Medicine, Mashhad University of Medical Sciences, Mashhad, Iran; 5 Pharmaceutical Research Center, Mashhad University of Medical Sciences, Mashhad, Iran

**Keywords:** Polymorphism, Renin angiotensin, Endothelial nitric oxide synthase, Premature cardiovascular disease

## Abstract

**Introduction:**

The study of polymorphisms and their relationship with diseases is very important for risk assessment. The aim of this study was to determine the relationship between early risk of coronary artery disease(CAD) with renin-angiotensin(RAS) genes and endothelial nitric oxide synthase(eNOS) in a sample of the Iranian population.

**Methods & materials:**

In this cross-sectional study, 63 patients with premature CAD and 72 healthy samples were enrolled. Polymorphism of the promotor region of eNOS- and ACE-I/D (Angiotensin Converting Enzyme-I/D) polymorphism was evaluated. Polymerase chain reaction (PCR) test and PCR-RFLP (Restriction Fragment Length Polymorphism) was performed for ACE and eNOS-786 gene, respectively.

**Results:**

The frequency of deletion(D) for the ACE gene was significantly higher in patients(96% versus 61%; P < 0.001). Conversely, the number of defective C alleles for the eNOS gene was similar in both groups (p > 0.9).

**Conclusion:**

ACE polymorphism seems to be an independent risk factor for premature CAD.

## Introduction

Despite of decrease in mortality by improvement of treatment options, one of the main etiology of death all over the world is still coronary artery disease (CAD) [18]. Many potential risk factors like environmental, biochemical, and inherited cause CAD due to progress in atherosclerotic lesions [7, 30]. Although there are some known genetic loci associated with CAD, less heritability of CAD is explained [10]. It seems that genetic risk factors have marked effect on etiology of CAD in young patients, because they have not enough time to expose to environmental factors [27]. Premature CAD is defined as: CAD in women younger than 55 years and men younger than 45 years, but this age range varies from 45 to 65 years in different studies [22].

Nowadays, the study on polymorphisms and their relationship with diseases is very important for risk assessment. Therefore, the analyzing of the association of any polymorphism in different population is specific to that groups and can be considered as a marker to determine the risk of disorder [28]. Many recognized polymorphisms have been shown to be associated with the risk of premature CAD. Although the list of genetic variants correlated with cardiovascular disease is still expanding, the genes of the renin angiotensin system (RAS) polymorphisms have been extensively studied to date. This is based on the fact that RAS has a great impact on the cardiovascular system and plays a key role in stimulating vascular smooth muscle cell proliferation, intimal fibrosis, inflammatory reactions, pro-thrombotic processes, and plaque calcification [11]. Numerous studies have shown polymorphisms of various components of the angiotensin-converting enzyme (ACE) system with the progression of CAD in some individuals and the efficacy of ACE inhibitors and angiotensin II receptor blockers [2]. Three common polymorphisms of different RAS genes have been commonly attributed to the risk of premature CAD or MI in previous investigations. These polymorphisms include insertion/deletion polymorphism in the angiotensin converting enzyme (ACE) gene, A1166C polymorphism in the ATR1 receptor gene (cell surface receptor for angiotensin II), and M235T polymorphism in the angiotensinogen gene [21]. One of the most important endothelial cell products is nitric oxide (NO), an important endothelium-dependent vasodilator mediator produced by endothelial cells from l-arginine. Nitric oxide also controls the function of angiotensin II in cardiovascular tissues, which itself regulates nitric oxide synthesis (10). Another notable gene would be the endothelial NOS gene. Nitric oxide (NO) is a molecule synthesized enzymatically from l-arginine (l-Arg) by three isoforms of NO synthase (NOS), i.e. neuronal NOS (nNOS), inducible NOS (iNOS), and endothelial NOS (eNOS) [19]. NO synthesized by eNOS is important for maintaining endothelial homeostasis. It mediates vasodilatation and suppresses smooth muscle cells proliferation. It also has an antioxidant effect. It promotes aggregation and proliferation of thrombocytes or inhibition of apoptosis of myocytes. Thus, polymorphisms within eNOS gene are suggested to be associated with the CAD development [1].

In this study, we evaluated the association of polymorphisms of renin angiotensin system and endothelial nitric oxide synthase genes with premature cardiovascular disease in an Iranian population.

## Materials and methods

This was a case-control study in which we enrolled patients with premature CAD. Patients who referred to the cardiology department of Ghaem and Imam Reza hospitals, Mashhad, during 2020–2021 were selected. ACE I/D and eNOS polymorphism was tested on 63 patients and 72 controls.

### Sample size

#### Eligibility

Inclusion criteria were the age of onset of CAD less than 50 years and at least 50% of obstruction in large arteries or one of the branches diagnosed by angiography.

#### Definitions

Arterial hypertension was defined as systolic blood pressure equal to or greater than 140 mmHg and diastolic blood pressure equal to or greater than 90 mmHg as well as patients treated with antihypertensive drugs.

Patients with a history of diabetes and fasting blood sugar above 126 mg/dl and also patients treated with insulin or oral hypoglycemic drugs were defined as diabetes mellitus.

#### Study design

**Fig. 1:**
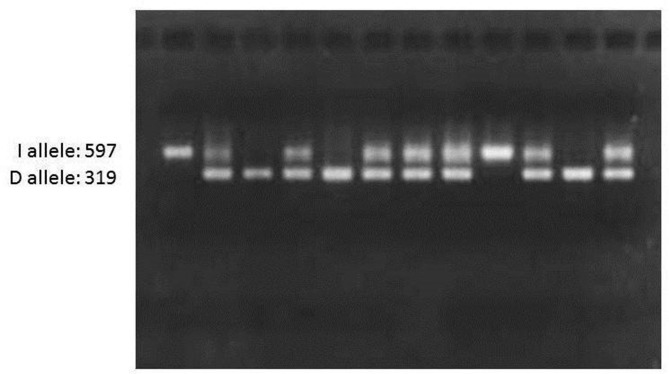
Agarose gel picture showing PCR products for NOS

**Fig. 2:**
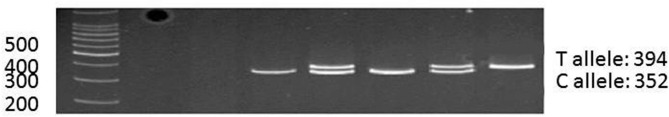
Agarose gel picture showing PCR products for ACE

For each patient, a checklist including demographic data, clinical risk factors (diabetes mellitus, hypertension, hypercholesterolemia, and smoking) and laboratory data (triglyceride, total cholesterol, LDL cholesterol, HDL cholesterol) were recorded. From the blood samples of the subjects, DNA extraction was performed by Kiagen kit. To evaluate the polymorphism of the studied genes, first the primer sequence of each gene was extracted from the relevant articles and their accuracy was confirmed by the NCBI site of the PRIMER BLAST section. PCR test was performed for ACE gene alone. For eNOS-786 gene, the PCR product was exposed to MboI enzymes at 37 ° C for 8 hours and then electrophoresed on 3% agarose gel and the bands related to each allele were examined. Genotyping of eNOS-786 region of endothelial nitric oxide synthetize enzyme by PCR amplification method with direct and inverse primers (sense: 5’-CACCCAGGCCCACCCCAACT-3 ‘; antisense: 5’-GCCGCAGGTCGACAGAGACT-3’) was performed according to the protocol of Esposti et al [12] (Fig. 1). PCR of ACE gene was done based on the methods described previously and using ACE primers (5’-GCCCTGCAGGTGTCTGCAGCATGT-3 ‘and 5’-GGATGGCTCTCCCCCGCCTTGTCTC-3’) based on the protocol of Lindpaintner et al [17] (Fig. 2).

#### Statistics

SPSS version 16 was used for statistical analysis in this study. Descriptive data are displayed as mean ± SD (mean ± SD). Genotype distribution and frequency of alleles were compared between the two groups using chi-square test. Freeman-Halton extensions associated with the Fisher exact test were used for alleles with a frequency of less than five. Logistic regression was used to determine the role of alleles in predicting disease. Significance level less than 0.05 was considered.

#### Ethics

This study was approved by ethical committee of Mashhad University of Medical Sciences (Ethical ID: IR.MUMS.MEDICAL.REC.1398.723). All steps of the study was in accordance with the Declaration of Helsinki.

## Results

A total of 135 patients (83, 61.5% male and 52, 38.5% female) entered the study. The mean ± SD age was 43.5 ± 4.3 years. Fifteen patients had diabetes (23.8%), 20 patients had hypertension (31.7%), 21 patients had hyperlipidemia (33.3%) and 11 patients were smokers (17.5%). Of them, 32% had one vessel involvement, 59% had two-vessel involvement and 9% had three coronary vessels involvement. Table 1 demonstrates the number and percentage of ACE, Dallel, Dhomozygote and eNOS-786 by study groups and statistical test results.

**Table 1:** The Comparison of ACE, D allel, Dhomozygote andeNOS in CAD and control groups

Characteristics		group		P value
		control	patient	
**ACE**	**DD**	10(13.9)	60(95.2)	< 0.001
	**II**	28(38.9)	3(4.8)	
	**D/I**	34(47.2)	0(0)	
**Dallel**	**no**	28(38.9)	3(4.8)	< 0.001
	**yes**	44(61.1)	60(95.2)	
**Dhomozygote**	**no**	62(86.1)	3(4.8)	< 0.001
	**yes**	10(13.9)	60(95.2)	
**eNOS**	**TT**	71(98.6)	62(98.4)	^*^>0.99
	**TC**	1(1.4)	1(1.6)	

*ACE = angiotensin converting enzyme; eNOS = endothelial nitric oxide synthase*

It is demonstrated that 95.2% (n = 60) of participants in the case group and 13.9% (n = 10) in control group had DD allele. Three patients (4.8%) and 38.9% (n = 28) of the control group had allele II. In the case group, no person had D/I but 47.2% of the control group had D/I. One person in each group had eNOS-786 of TC type in which the difference was not statistically significant (P > 0.99). Table 2 shows the number and percentage of variables of diabetes, hypertension, hyperlipidemia and smoking and the results of statistical tests.

**Table 2:** Investigating the relationship between gender, diabetes, high blood pressure, hyperlipidemia and smoking with the number of stenotic coronary arteries

Variable		SVD	2VD	3VD	P
**Gender (N, %)**	Male	15(44.1)	14(41.2)	5(14.7)	0.008
	Female	5(17.2)	23(79.3)	1(3.4)	
**DM (N, %)**	No	15(31.3)	29(60.4)	4(8.3)	0.748
	Yes	5(33.3)	8(53.3)	2(13.3)	
**HTN (N, %)**	No	11(25.6)	29(67.4)	3(7)	0.078
	Yes	9(45)	8(40)	3(15)	
**HLP (N, %)**	No	12(28.6)	27(64.3)	3(7.1)	0.435
	Yes	8(38.1)	10(47.6)	3(14.3)	
**Smoker (N, %)**	No	15(28.8)	32(61.5)	5(9.6)	0.552
	Yes	5(45.5)	5(45.5)	1(9.1)	

*SVD = single vessel disease; 2VD = two vessel disease; 3VD = three vessel disease; DM = diabetes mellitus; HTN = hypertension; HLP = hyperlipidemia*

Table 3 shows the number and percentage of ACE and eNOS-786 B parameters. In both groups of the ACE gene DD and II alleles, most individuals had two vessels disease. Subjects with one vessel disease were almost identical in both groups, but those with three-vessel disease were only in the D allele group. There was no significant difference between the two groups in terms of the number of involved vessels (P > 0.99). In the eNOS-786 TC group, there was only one subject who had three vessels’ involvement. There was also no significant difference between the two groups in terms of the number of stenotic arteries (P = 0.095).

**Table 3:** Comparison of ACE, Dallel, D homozygote, ENOS according to the number of closed vessels

Variable		SVD	2VD	3VD	P value
ACE	DD	19(31.7)	35(58.3)	6(10)	0.99
	II	1(33.3)	2(66.7)	0(0)	
eNOS	TT	20(32.3)	37(59.7)	5(8.1)	0.095
	TC	0(0)	0(0)	1(100)	

*ACE = angiotensin converting enzyme; eNOS = endothelial nitric oxide synthase*

## Discussion

RAS plays an important role in the pathophysiology of heart diseases such as coronary artery stenosis. Coronary artery stenosis is a polygenic disease and its occurrence and severity depends on environmental and genetic factors. The association of RAS polymorphism with classical risk factors for CAD such as hypertension, obesity, diabetes and hyperlipidemia has been reported. ACE I / D gene polymorphism is a strong risk factor for CAD. In this study, patients with premature CAD and healthy control group were evaluated to determine the relationship between CAD and RAS gene and eNOS-786 polymorphism in a sample of the Iranian population. Although available data show that assessment of a single gene role in the development of CAD is insufficient, most case–control studies focus to analyze only one genetic polymorphism in regarding to the disease. To draw meaningful conclusions in the field, several polymorphisms with specific gene–gene as well as gene-environment interactions should be studied. This has been confirmed by earlier researches [27]. We found that there is a strong association between DD polymorphism and CAD. According to genotype studies, DD is associated with MI, hypertrophic cardiomyopathy and coronary artery occlusion, as well as other heart diseases. DD genotype is more closely related to CAD than the other two genotypes (II and ID). The DD genotype of the ACE gene has been identified as a risk factor for heart disease in other communities, such as the Caucasus, Australia, and China [2]. In another study in Saudi Arabia examining of diabetic patients showed that there is a clear correlation between ACE DD and CAD [3].

Some other previous investigations worldwide [2, 4, 9, 11, 14], demonstrated that there is a significant correlation between ACE polymorphism and CAD. RAS gene polymorphism is not only an independent risk factor for CAD progression, but also can affect other CAD risk factors such as hypertension, atherosclerosis and thrombosis. The result is an increased risk of CAD in people who carry these polymorphisms [24]. Conversely, another study in Brazil, in patients with obstructive lesions, there was no association between ACE gene polymorphism and CAD in both Caucasian and Brazilian African breeds [5]. Rallidis et al. found no association between RAS gene polymorphism and premature CAD in a small sample size of patients with early MI [23]. On the other hand, in an investigation in United Kingdom on 684 patients with acute MI and 537 control people, there was no association between ACE I/D polymorphism and MI risk [26]. In another study, the relative risk of the D allele was estimated 1.07 for ischemic heart disease and 1.05 for MI, so the ACE genotype does not significantly increase the risk of ischemic heart disease and MI [17]. Studies support the idea that in the larger statistical population there is an association between RAS gene polymorphisms and CAD and early MI [23].

Our results showed that there was no significant relationship between eNOS-786 polymorphism and CAD. There was no association between the type of polymorphism in eNOS-786 gene and CAD in younger patients in previous studies [1, 15, 20, 29]. In the study by Zigra et al. [31] no association was found in analyzing the whole CAD group. However, the authors demonstrated that prevalence of TT homozygotes were significantly more common only in patients with ‘normal’ or ‘near normal’ coronary arteries compared to controls [31]. In turn, a strong significant association between one of eNOS-786 genotypes and the disease was found by Cam et al. [6]. They showed that NOS3 TT genotype was an independent risk factor for premature CAD with OR = 15.35. Saini et al. [25] observed that mean NO level was significantly lower in subject with GT genotype than in those bearing GG genotype. However, the mean NO levels in the CAD patients with GT genotype were lower comparing to the control group. According to authors, the T allele of 894GT polymorphism in NOS3 gene may be a marker for endothelial dysfunction which is characteristic for CAD. Kim et al. found no association between eNOS G894T and CAD risk [16]. Similarly, Granath et al., in a Caucasian Australian population with premature CAD and younger than 50 years, showed that there was no significant relationship between any of the eNOS-786 gene polymorphisms and early CAD in this population [13]. However, some studies have shown an association between this polymorphism and the risk of CAD. Colombo et al. studied 451 people and showed that the T786C-eNOS gene polymorphism could significantly increase the chance of developing CAD in Italian populations [8]. The discrepancy between the results obtained in the present study and other researches can be attributed to differences in the ethnic and racial backgrounds of the participants in our study with other populations. In addition, environmental factors and the interaction between genes and the environment can also contribute to these differences.

## Conclusion

The carrier-state of DD allele of RAS polymorphism was associated with premature CAD but eNOS-786 was not. Recognition of this polymorphism in different populations could help healthcare policy makers to manage the burden of cardiovascular disorders, logically. Performing large-scale study in different regions of Iran could be helpful to mapping the distribution of polymorphism and programming for their future treatments.

## Data Availability

Data are available from the authors upon reasonable request and with permission of the corresponding author.

## Abbreviations

RAS: Renin angiotensin
eNOS: Endothelial nitric oxide synthase
ACE: Angiotensin Converting Enzyme
I/D: Insertion/ Deletion
PCR: Polymerase chain reaction
RFLP: Restriction Fragment Length Polymorphism
CAD: Coronary artery disease
MI: Myocardial infarction
NO: Nitric oxide
l-Arg: l-arginine
nNOS: Neuronal NOS
iNOS: Inducible NOS
